# Pseudoangiomatous stromal hyperplasia – a benign and rare tumor of the breast in an adolescent: a case report

**DOI:** 10.1186/s13256-017-1426-9

**Published:** 2017-10-05

**Authors:** Alberto Testori, Marco Alloisio, Valentina Errico, Edoardo Bottoni, Emanuele Voulaz, Bethania Fernandez, Stefano Meroni, Matilde De Simone, Ugo Cioffi

**Affiliations:** 1 Division of General and Thoracic Surgery, Humanitas Research Hospital, Via Manzoni, 56, 20089 Rozzano, Milan Italy; 2 Division of Anatomopathology, Humanitas Research Hospital, Rozzano, Milan Italy; 3 Division of Breast Imaging, European Institute of Oncology, Milan, Italy; 4 Department of Surgery, University of Milan, Milan, Italy

**Keywords:** Pseudoangiomatous stromal hyperplasia (PASH), Mesenchymal breast neoplasm, Benign breast disease

## Abstract

**Background:**

Pseudoangiomatous stromal hyperplasia is an uncommon mesenchymal breast neoplasm.

**Case presentation:**

Here we present a case of an 11-year old hispanic girl affected by bilateral mammary nodular pseudoangiomatous stromal hyperplasia, an uncommon breast disease, with a review of the literature related to diagnostic workup, differential diagnosis, and management. A rapidly growing mass in the breast may be stressful for both parents and child as the suspicion of malignancy arises. Multiple wide excisions of both breasts were performed.

**Conclusions:**

The purpose of this case report is to draw attention to the fact that most emerging lesions of the breast in girls during puberty are benign diseases.

## Background

Breast masses are uncommon in children and adolescents. The majority of them are benign tumors like fibroadenomas [1–3] or are associated with inflammation due to infection. Mammary pseudoangiomatous stromal hyperplasia (PASH) was first described by Vuitch *et al*. in 1986 [4]. PASH is a rare benign proliferating breast condition and it usually presents as a fast-growing palpable lesion in women between 30 and 40 years of age and is exceptionally rare in adolescents. There have been only a few reported cases of bilateral PASH in girls. Surgical resection remains the gold standard of treatment.

We report a rare case of an adolescent girl who presented with a bilateral breast PASH that occurred just after menarche and showed rapid growth over a short time period. This case is a diagnostic and therapeutic challenge, taking into account our patient’s age and the controversial treatment recommendations. Good collaboration between surgeons and pathologists is essential for an accurate diagnostic process and aims to avoid overtreatment.

## Case presentation

**Fig. 1:**
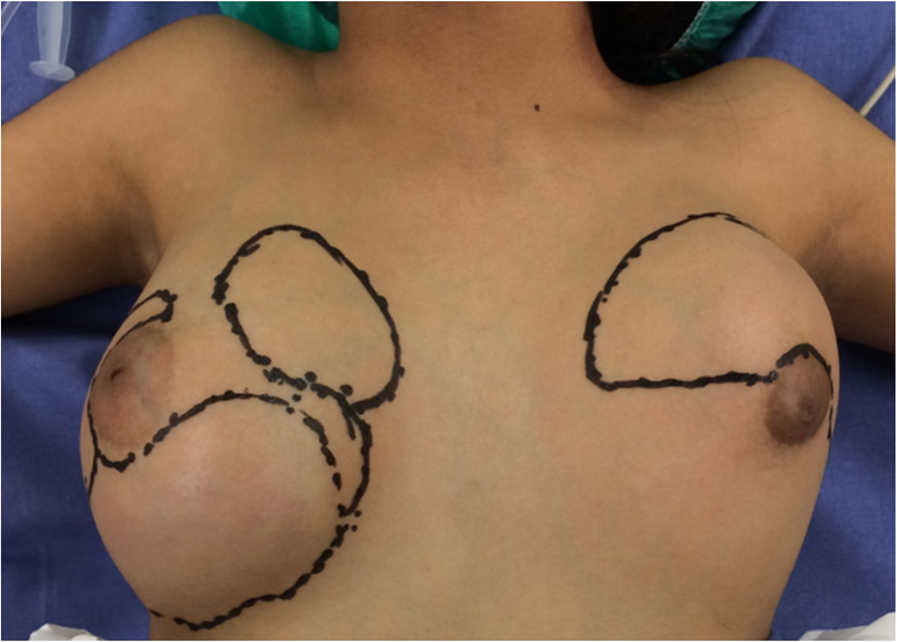
Preoperative view of the patient showing asymmetrically enlarged breasts

**Fig. 2:**
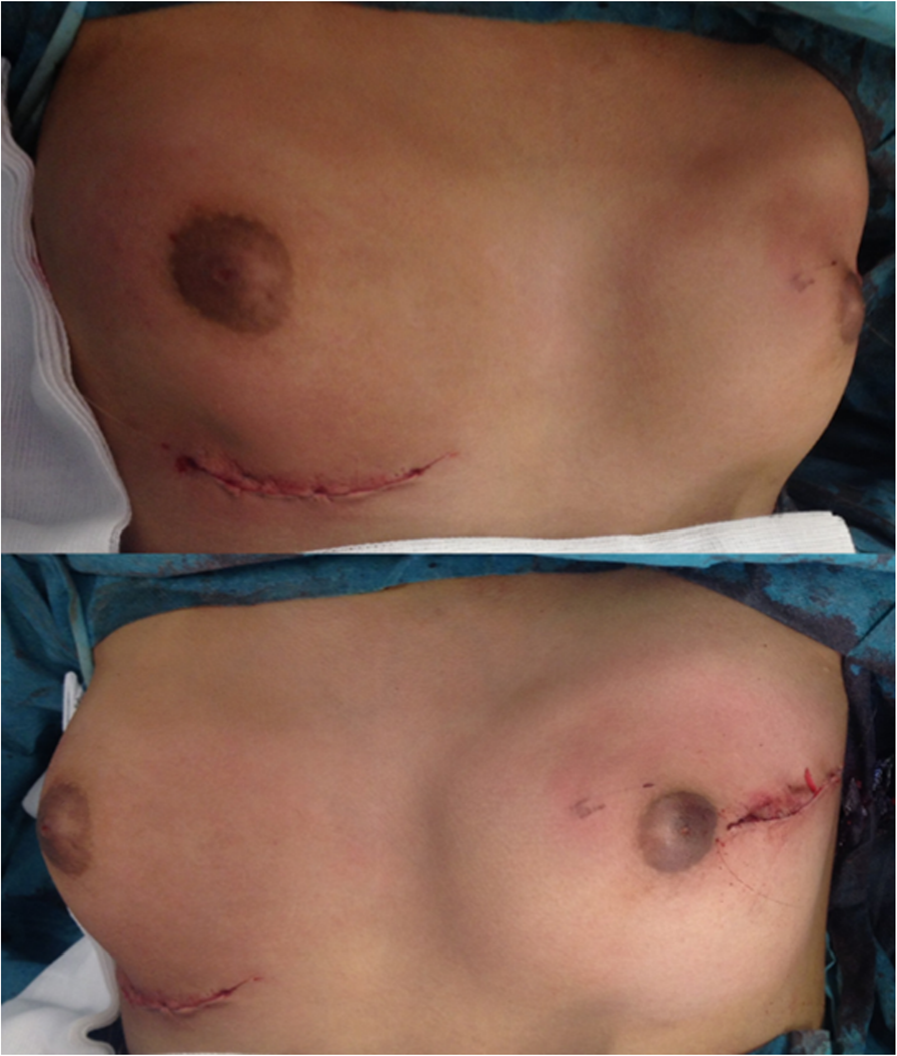
Postoperative view after bilateral multiple wide excision

An 11-year old hispanic girl presented with a rapidly enlarging and painful mass in both breasts. She had no family history of breast disease or breast and ovarian cancer. A physical examination showed large solid lumps with a well-circumscribed border in both breasts measuring 8 cm and 5 cm in the right and left breast, respectively (Figs. 1 and 2).

An ultrasound examination was performed showing many solid oval-shaped nodules, well-defined margins, and a homogenous hypoechoic mass like a fibroadenoma. No cystic collections were appreciated within the nodules and the lesions were mildly vascularized at a color-Doppler evaluation. There was no acoustic shadowing, bilaterally, and no sonographic sign of adjacent tissue invasion. There was no axillary lymphadenopathy. The ultrasound findings were not considered suspicious for malignancy; the diagnosis was oriented to a benign etiology such as fibroadenoma, PASH, or hamartoma.

Taking into account our patient’s age, clinical presentation, and tumor size, a multidisciplinary team recommended multiple wide surgical excisions not to be followed by adjuvant therapy. Wide excisions of her breasts were performed for complete removal of the nodules. During surgery, both her breasts were found to be studded with nodules of varying sizes with little normal breast tissue. The final pathologic diagnosis was bilateral mammary nodular PASH with some juvenile fibroadenoma.

**Fig. 3:**
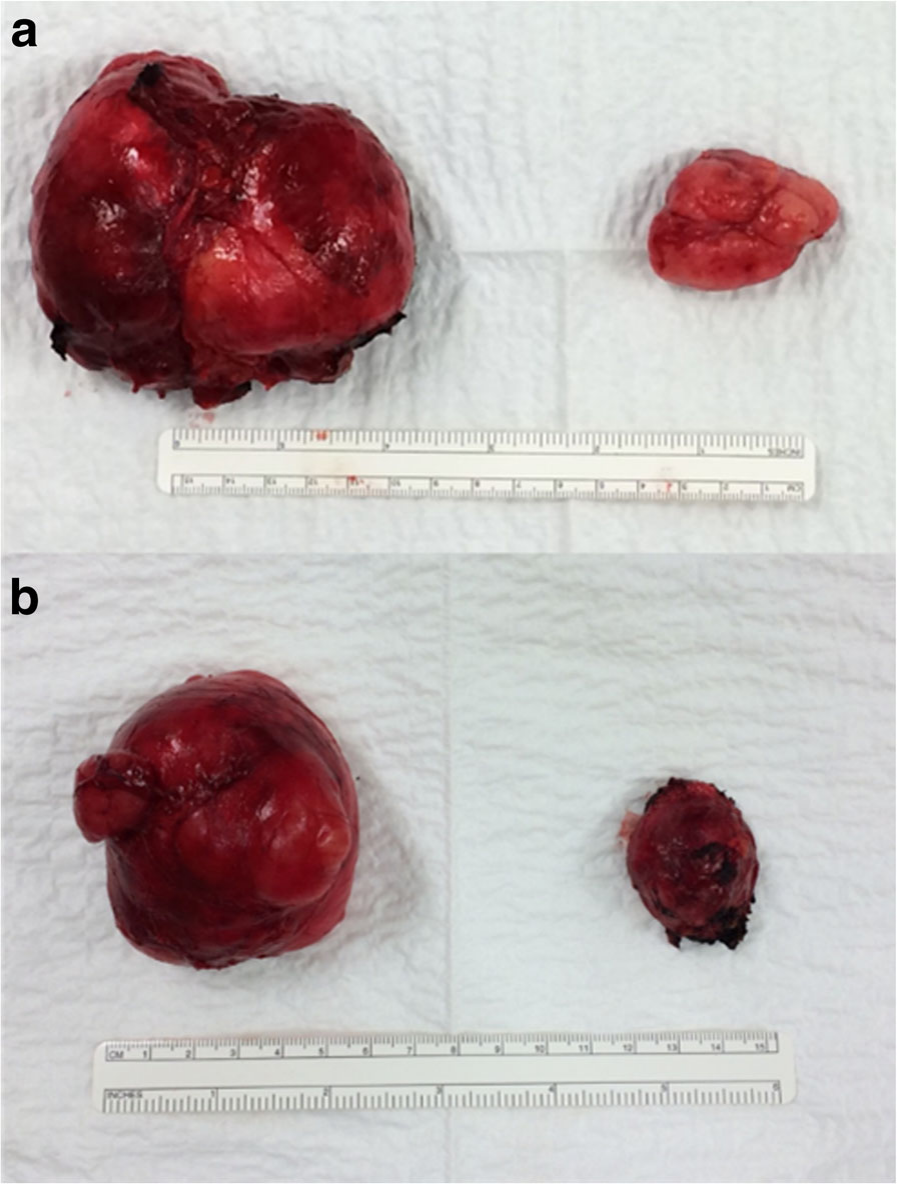
The gross image of the right (**a**) and left (**b**) mammary masses shows well-demarcated, rubbery-firm, tan-brown external surfaces

**Fig. 4:**
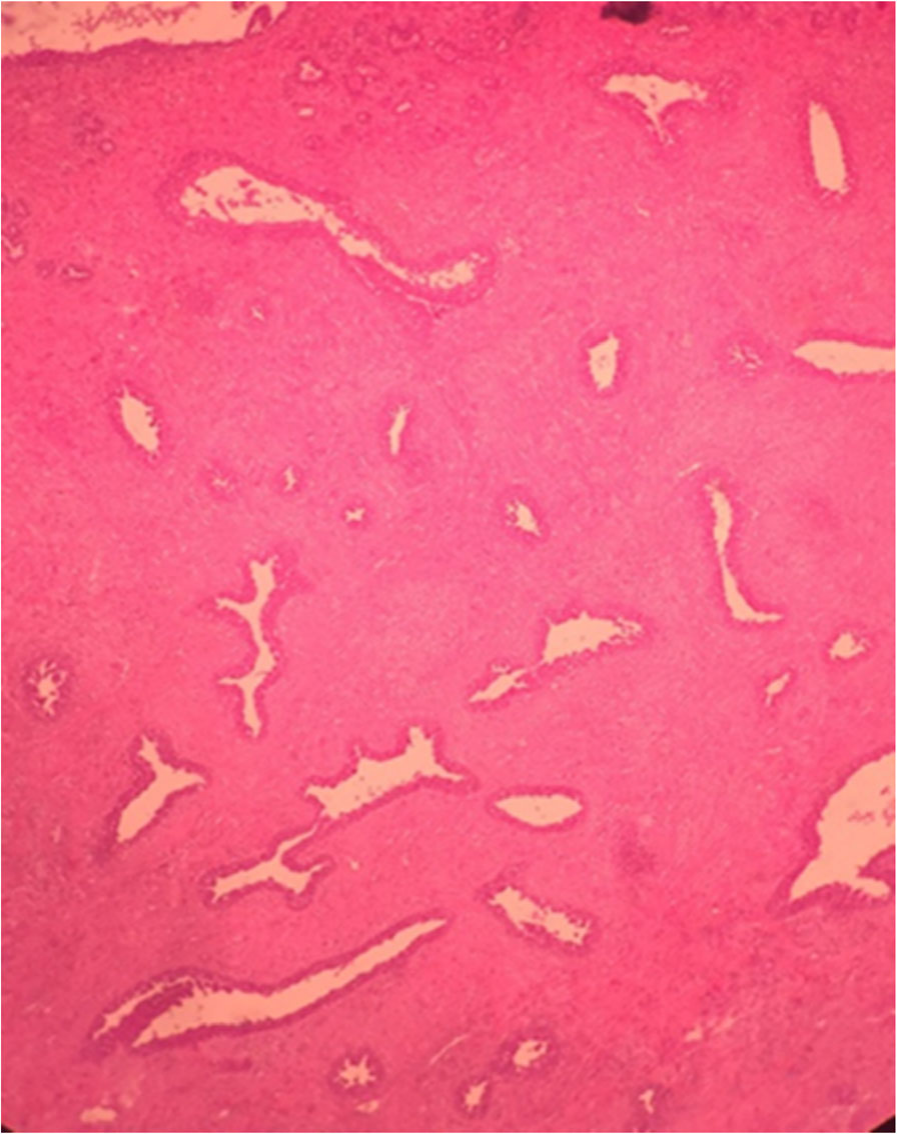
The fibroadenoma (×10) at histology

**Fig. 5:**
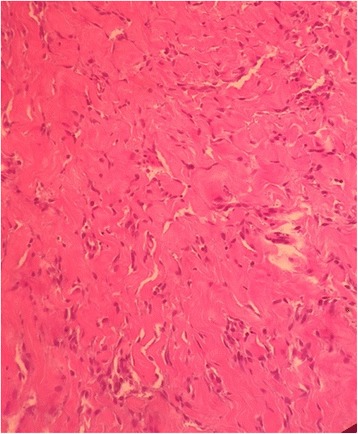
Some focal aspects of pseudoangiomatous stromal hyperplasia (× 20) at histology made of complex interconnected slit-like channels lined by spindle cells in collagenous stroma

In order to study tissue with a light microscope, the specimen was fixed in formalin (10%), cut into sections of 5 μm and then stained with hematoxylin-eosin. A histopathological examination of the lesion showed anastomosing slit-like pseudovascular spaces in PASH nodules. The external surfaces of the PASH nodules showed a well-demarcated, rubbery-firm, tan-brown appearance (Fig. 3). At histology (Figs. 4 and 5), a fibroadenoma (Fig. 4) was observed with some focal aspects of PASH made of complex interconnected slit-like channels lined by spindle cells in collagenous stroma (Fig. 5). There was no evidence of atypia, mitosis, or pleomorphism.

At her 3-year follow-up, she was asymptomatic with no evidence of recurrence. She was submitted to routine follow-ups in view of local recurrence. She was followed-up with clinical and ultrasound examinations.

## Discussion

Usually, adolescent breasts grow rapidly soon after the first menstrual period. Among breast masses in adolescent girls, some pathologic lesions such as giant fibroadenoma, phyllodes tumor, PASH, juvenile papillomatosis (Swiss cheese disease), and virginal breast hypertrophy (juvenile macromastia) rapidly and massively increase in size over a short time period. The clinical presentation as a rapidly enlarging breast mass, as seen in our patient, is extremely rare and may therefore raise suspicion of malignancy. Other less common causes are lipoma, mammary hamartoma, breast abscess, fibrocystic change, and adenocarcinoma but PASH should always be considered in adolescent girls presenting with a rapidly enlarging breast mass.

Although its prevalence is difficult to accurately estimate, tumor-forming PASH is an uncommon mesenchymal neoplasm of the breast and typically affects women in the reproductive age group; it is extremely rare in an adolescent girl, such as in our case report.

PASH is frequently an incidental histologic finding in breast biopsies performed for other reasons [5]. Sometimes, it can present as a firm, painless breast mass, which has been referred to as nodular PASH, such as our case.

It is hypothesized that PASH originates from myofibroblastic cells with a variable expression of myoid and fibroblastic features demonstrating stromal proliferation; it has the appearance of anastomosing slit-like pseudovascular spaces lined by spindle-shaped cells. The exact etiology is unknown, but hormonal etiology could be one cause. In fact, intense patchy staining of progesterone (PR) was noted in the nuclei of stromal cells in cases of PASH, whereas estrogen (ER) expression was more variable and faint. So, the main hormone implicated to stimulate the myofibroblasts is PR. In contrast, the stromal cells in uninvolved breast tissue did not show any expression of either ER or PR [6, 7].

As described in the literature, the fact that more than half of postmenopausal women with PASH tumors received hormone replacement therapy supports the role of hormonal influence in its pathogenesis [6–8]. This concept is supported by a previous case report of rapid growth of PASH during pregnancy [9]. By contrast, our case and the few reports in the literature of adolescent girls with PASH do not support hormone replacement therapy.

Given the possible etiological role of hormones, antihormonal therapy could theoretically serve as an alternative noninvasive approach in the management of PASH tumors. Successful cases in which patients respond at least partially to tamoxifen therapy have been reported [10, 11].

In our case, the ultrasonographic findings of nodular PASH were indistinguishable from those reported for juvenile fibroadenoma and for phyllodes tumor. Thus, histological study was required for the definitive diagnosis. Only skilled pathologists should perform differential diagnosis. The pathologist should take this neoplasm into consideration due to differential diagnosis of stromal myofibroblastic proliferation; nodular PASH has the appearance of anastomosing slit-like pseudovascular spaces lined by spindle-shaped cells.

The differential diagnosis of a large breast mass in adolescent females is important for determining treatment modalities. The most important differential diagnosis on histopathological examination is angiosarcoma. PASH has an excellent prognosis, with very low risk of recurrence and it does not require any additional specific treatment when it is diagnosed incidentally from a specimen.

Treatment strategies for PASH remain controversial. Wide surgical resection or mastectomy is requested when there is an important mass-effect by PASH, whereas other cases may only require local excision or conservative therapy. Wide excision without lymphadenectomy remains the cornerstone of management of PASH. However, some cases with diffuse involvement or multiple recurrences may necessitate mastectomy [6].

In the breast diagnostic work-up, sonography and fine-needle aspiration or core biopsy are used after physical examination. In our opinion, imaging techniques are not fundamental in obtaining a diagnosis of rapid growth PASH in adolescent girls because the final diagnosis is determined by surgical approaches (wide resection or quadrantectomy or mastectomy). In fact, some lesions can often be ruled out by physical examination or by typology of presentation (for example lesion in rapid growth).

Considering the age of our patient, and in order to prevent psychological damage, we decided not to perform any interventional procedures for a preoperative diagnosis, considering the large size of the bilateral masses, the rapid growth, and the surgical indication. We did not perform mammography for the obvious radioprotection reason.

A multidisciplinary team recommended multiple wide surgical excisions not to be followed by adjuvant therapy; in particular, the lack of evidence and our patient’s age did not support antihormonal therapy.

## Conclusions

We can confirm that PASH tumors must be treated with local surgical excision with clear margins; the prognosis is excellent, with minimal risk of recurrence after adequate surgical excision.

Collaboration between surgeons and pathologists is essential for an accurate diagnostic process and aims to avoid undertreatment or overtreatment.
